# The moral imperative to approve pregnant women’s participation in randomized clinical trials for pregnancy and newborn complications

**DOI:** 10.1186/s13010-019-0081-8

**Published:** 2019-09-06

**Authors:** Dan Kabonge Kaye

**Affiliations:** 1 College of Health Sciences, Department of Obstetrics and Gynecology, Makerere University, P.O. Box 7072, Kampala, Uganda; 2 Berman Institute of Bioethics, Johns Hopkins University, 1809 Ashland Avenue, Baltimore, 21205 USA

**Keywords:** Clinical trials, Pregnancy, Fair inclusion, Clinical research, Moral imperative, Doctrine of double effect

## Abstract

**Background:**

There is longstanding consensus on the need to include pregnant women in research. The goal of clinical research is to find highly regulated, carefully controlled, morally responsible ways to generate evidence about how to effectively and safely prevent illness or treat sick people. This manuscripts present a conceptual analysis of the ethicality of clinical trials in 3 scenarios: where the pregnant is involved in clinical trials as a participant during pregnancy for data that addresses pregnancy complications, where the pregnant woman consents to clinical trial participation for an unborn baby that has complications, to generate data on complications at this stage of life, and where the mother may consent for participation of their newborn child in clinical trials.

**Methods:**

Conceptual analysis.

**Findings:**

Investigators often choose to exclude pregnant women and newborns from research, even where there is possibility for them to benefit from the study intervention. Objections include vulnerability of pregnant women, altered pharmacokinetics and risk of adverse effects, with a need to balance potential maternal and fetal risks and benefits of research participation. While the objections may be valid, not performing research magnifies what should be a carefully controlled risk during research, pushing this risk into the clinical setting, and subsequently posing a challenge to clinicians who are faced with making treatment decisions for pregnant patients with limited evidence of efficacy and safety. The potential benefits of fair inclusion in clinical trials outweigh the potential risks.

**Conclusion:**

Research involving pregnant women is necessary to provide women with effective treatment during pregnancy, to promote fetal safety (such as by avoiding the clinical use of drugs that may be harmful to the developing fetus), and to reduce avoidable harm from suboptimal care (such as from underdosing) and to provide pregnant women, their fetuses and newborns (with access to potential benefits of research participation).

## Introduction

Traditionally, there are arguments as to whether it is ethical to recruit pregnant women into research [1–3]. There are several situations which may require a pregnant woman to participate in research or to consent for the participation of her newborn. The clinical trials may seek to address the management of pregnant women for complications of pregnancy or childbirth, or may seek to recruit women without complications, where the unborn baby has complications or the newborn at birth is likely to suffer from foreseeable complications. This manuscript concerns maternal decision-making about participation in clinical trials for complications in pregnancy or newborns (where the mother is the research participant but it is the mother, fetus or newborn at risk or affected by the complication). However, my arguments extend to research designed to generate data primarily for the mother as a participant or similar mothers, as well as to research designed to address complications in the newborn (where the mother makes the informed decision to for the fetus or newborn to participate in clinical trials.

Those who argue against recruiting pregnant women or newborns in research base their reasons on two premises. First is the ethical concern of equity. Fair subject (participant) selection which refers to fair recruitment and enrollment practices is one of the seven ethical requirements for clinical research to be ethical [4]. Founded on the ethical principle of justice, this ethical requirement holds that “*particular individuals, groups or communities should neither bear an unfair share of the direct burdens of participating in research, nor should they be unfairly excluded from the potential benefits of research participation*.” [4]. Secondly, women in general and pregnant women in particular were excluded from clinical trials on the assumption of heterogeneity (related to hormonal cycles and other sex-based variables that might impact the medical conditions under study). Thirdly, due to reasons (or concerns) of vulnerability (potential risks or harms related to research), pregnant women, unborn babies or the neonates are traditionally excluded from most research. This uncertainty of whether, when and which research is ethically permissible during pregnancy (for maternal or fetal complications) and in newborn (for neonatal complications) is compounded by lack of data about what potential harms pregnant women, unborn babies or newborns can safely be exposed to during research. This necessitates that investigators take additional steps to enroll women to guarantee protection from harm them by ensuring exposure to no greater than minimal risk [5]. This paper presents a conceptual analysis of the ethicality and moral imperative to permit the participation of pregnant women and newborns in clinical trials for pregnancy or newborn complications.

## Main text

There is longstanding consensus on the need to include pregnant women in research. In 1994 an Institute of Medicine report [6] on challenges and barriers to the inclusion of women in clinical research recommended that pregnant women be presumed eligible for participation in clinical studies. The majority view in the report recommended that investigators and Reserch ethics committees exclude pregnant women from participation only when (1) there was no prospect of medical benefit to the pregnant woman, and (2) a risk of significant harm to the offspring was known or could be plausibly inferred. Despite this, pregnant women continue to be excluded from therapeutic or preventive trials. Yet clinical trials remain the golden standard for drug evaluation. For newborn participation in clinical trials, parents take the responsibility for their child’s recruitment into research. Obtaining parental consent necessitates providing the necessary information to help parents to make an informed decision, after weighing the potential benefits and risks of the neonate’s participation [1]. Though significant challenges exist in when and how this disclosure of information should occur, there is consensus that this is a necessary step for ethical inclusion of mothers or their newborns in research. Failure to provide important information (or failure to comprehend the disclosed information) may lead to difficulties in decision-making process of the pregnant women, failure to recruit adequate numbers of mothers or newborns in research, or untimely discontinuation of pregnant women or their newborns from research participation [1–3]. Appropriate explanation of the relevance and rationale of the randomisation process to parents is particularly important in order to help them make informed decisions for the neonate’s participation [3].

The goal of clinical research is to find highly regulated, carefully controlled, morally responsible ways to generate evidence about how to effectively and safely prevent illness or treat sick people. For pregnant women as for any other population, ‘sick women get pregnant and pregnant women get sick’ [5]. Therefore, not performing research with pregnant women just magnifies what should be a carefully controlled risk during research and pushes it into the clinical setting. This poses a challenge to clinicians who are faced with making treatment decisions for pregnant patients with limited evidence of efficacy and safety [5]. Consequently, investigators choose to exclude pregnant women and newborns from research, even where there is possibility for them to benefit from the research.

### Concerns regarding inclusion of pregnant women in research

Certain research, particularly, non-therapeutic research, may have no prospect of medical benefit to the pregnant woman, the progression of pregnancy or the fetus, thereby posing unnecessary and avoidable harm [5–7]. Other research, though beneficial to the mother and fetus, may pose a risk of significant harm to the fetus [6]. Considering that many substances, including medications, can cross the placenta and potentially irreversibly affect fetal growth, structure, or function, there might be significant risk of harm to the unborn baby [6]. The foremost concern for providers and mothers is the safety of the medication for the mothers and the fetus [7–9]. Also, the validity of the studies may be affected by the significant physiologic changes in nearly all body systems during pregnancy, among which are doubling of maternal blood volume, alterations in binding proteins, changes in pharmacokinetics and bioavailability of drugs in pregnancy and alteration in metabolism and excretion [4–6]. Besides, toxicity and teratology studies of pregnant animals imperfectly or inconsistently predict human effects, even for drugs whose teratogenicity and toxicity profile may be acceptable from prior animal studies [9]. Similarly, there may be inadequate information to determine whether potential benefits of new medications exceed the unknown (teratogenic) risks for many medications [8, 9]. For these reasons, pregnant women and newborns may continue to be excluded from pharmacological therapeutic or preventive clinical trials.

### Physiological complexity in pregnancy

Pregnancy combines both physiological and ethical complexity [6]. The physiological complexity is related to the physiological changes that affect all organs and systems in the body, though to different extents, as well as the need to avoid preventable harm. During pregnancy, physiologic changes occur in nearly all organ systems, virtually assuring that the pharmacokinetics (PK) of drugs administered to pregnant women will be impacted [10, 11]. Drug PK are different in pregnancy and non-pregnancy state, due partly changes in blood and plasma volume and difference in expression and availability of genes that code transporter and carrier proteins in this state [12]. Logistically, PK trials are challenging to conduct in pregnancy and most available data is from limited and “opportunistic studies” performed when pregnant women are already receiving a therapeutic agent [9]. Though there are significant physiologic changes in pregnancy, PK and efficacy of drugs used in pregnancy remains largely unknown. Ideally, every medication should be studied in each trimester of pregnancy and during the postpartum period, to allow for dosing adjustments in pregnancy to minimize toxicity while ensuring efficacy [11]. Dosing recommendations for pregnant women are usually extrapolated from studies in nonpregnant patients, and most medications prescribed in pregnancy are used “off-label” [13, 14]. Pregnancy changes affect drug pharmacodynamics (distribution, binding, absorption, metabolism, and excretion of drugs), and thus may impact their pharmacodynamic properties during pregnancy. There is danger in not knowing [15].

The ethical complexity is reflected in the need to balance the interests of the pregnant woman and the fetus [5]. Pregnancy doesn’t alter a woman’s capacity for autonomous decision-making, and a pregnant woman is capable of making complex medical decisions for herself and her fetus that reflect her personal, family or social values [5]. The first concern is in aligning maternal and fetal interests. Maternal and fetal interests usually align, as appropriate care of the woman is necessary for the health of the fetus. These interests may diverge in the setting of research, especially where research is not focused on concerns of pregnancy, labor, or fetal health [5]. While including pregnant women in the study of new drugs potentially could cause fetal harm, it is critical to recognize that excluding pregnant women from research also can lead to harm [5]. The exclusion of pregnant women from research participation is a serious ethical problem because of the potential harms that women and their fetuses may suffer consequent to lack of knowledge about the drugs, nutrients and vaccines used during pregnancy [5]. There are many women who conceive when they are on long-term medication, especially from unintended pregnancy [5]. Others may fall sick, and therefore require medication during pregnancy. Therefore, there is a need to generate information through research on the effectiveness of medications that address medical problems that may occur in pregnancy or may be specific to pregnancy [16–19]. Besides, over 80% of pregnant women may receive drugs that have not been evaluated for safety in pregnancy [18].

The second concern is that pregnant women are vulnerable, and there is potential risk of harm or exploitation as a vulnerable group [17]. This view considers appropriate inclusion in clinical trials for different classes of persons, such as infants, children, women or pregnant women. However, it is widely accepted (in principle and often in practice) by researchers, research sponsors, research ethics committees and research regulators, that research involving children, women in general and pregnant women in particular is ethical if it is intended to primarily benefit these populations and has high possibility of potential benefit [20–24]. There is the additional presence of a fetus, who may be harmed by the investigative products being tested. Yet pregnant women and their fetuses deserve timely access to safe, effective, evidence-based care and should be included in clinical trials (where they stand to benefit, such as for drugs and vaccines), unless when there is a compelling scientific or ethical reason not to do so [19, 23].

The third reason is the risk of harm from altered pharmacokinetics and pharmacodynamics of drugs, which may lead to inadequate bioavailability of the prescribed medication. Pharmacokinetic differences between pregnant and non-pregnant women affect drug bioavailability [10, 11]. Sometimes the pharmacokinetic parameters increase, sometimes they decrease, and sometimes they stay the same, suggesting that neither intuition nor clinical experience may be trusted [10, 11]. Where treatment of the mother is inadequate, the fetus is exposed to therapies at a dose which does not provide a benefit to the mother [19]. Pregnant women and their unborn fetuses are a population with heightened risks from infectious conditions and many pregnant women receive antimicrobials during pregnancy for various indications [25]. However, many of the available antimicrobials in current use have inadequate data to fully inform evidence-based dosage recommendations or on safety, efficacy and fetal risk [25].

### The ethical complexity of RCTs in pregnancy

Even where opportunity to participate in randomized clinical trials (RCTs) is offered, there are challenges in recruiting pregnant women as participants in RCTs [26]. Many women assessed for eligibility may not be recruited in the research, yet high assessed-but-not-recruited rates affect the feasibility (from participant-related, clinical trial-related or investigator-related biases) and external validity of conducting obstetric trials [26]. There are also challenges in designing the studies. Small, well-designed Phase I safety trials for pregnant women should be initiated at the same time to begin at the same time as Phase III efficacy trials in the general population so that trials that recruit women are initiated only when the investigational product has successfully completed Phases I and II in (the general population of) men and non-pregnant women [16]. The second option is designing Phase I trials for women that are nested in late Phase II or Phase III trials, with enhanced monitoring for pregnant women [16].

Despite the increasing recognition of profound gaps in research on the safety and efficacy of drugs often prescribed to pregnant women, and calls questioning the practice of routinely excluding pregnant women from research participation, the regulatory requirements are difficult to achieve [27]. A major barrier is consideration for the safety of the fetus. There is a valid argument, however, that even many of the drugs currently used by pregnant women may in fact be unsafe for the fetus [5]. Pregnant women may inadvertently consume unsafe or dangerous medications, or may avoid needed drugs, both of which outcomes are potentially harmful to pregnant women and their fetuses [28]. Data on safety from the general population may not be adequate or comprehensive. For instance, data was available on contraindications of ACE inhibitors in the second and third trimesters but no data was available for use in the first trimester until a 2006 report [29] linked the drug to increased risk of fetal cardiovascular and neurological abnormalities.

Another barrier for medical investigators is the risk of adverse outcome in the fetus or mother. Medications can cross the placenta and irreversibly affect fetal growth, structure, and function if the fetus is exposed at a critical stage during its development [5]. In contemplating research participation, just like in therapy, the overarching concern (for both providers and research participants) is safety of medication for the fetus. However, environmental, nutritional and other health factors during pregnancy can affect fetal growth and development. This risk increases reluctance in the research community to include pregnant women in clinical investigations. However, this conservative stance enhances neither fetal nor maternal safety. Besides, there is evidence that this risk to the fetus may be overestimated in some studies [30] and that potential benefits may far outweigh this risk in other studies such as gene transfer research [21].

Lactation is another critical consideration for using investigational products in pregnant women who are research participants. For pregnant women close to term, there is need to assess safety of investigational products during lactation [31]. While most medications can be taken safely during breastfeeding, the potential risks of infant toxicity do exist because all medications (to some extent) are excreted into breast milk [31]. The extent to which secretion occur depends on within-drug variation (such as dosing), between-drug variation (such as chemical characteristics of the medication) and host factors (such as gestational age, maternal pharmacokinetics during pregnancy or puerperium and drug binding and metabolism) [31]. It is important for rational risk assessment to balance the toxicity risk from breast milk secretion against the potential benefits (of the research) and lactation, as the health potential benefits of both mother and child are significant.

### The regulatory environment is not supportive for research on pregnant women

Among the different reasons for the continuous underrepresentation of pregnant women in research is the problem that guidelines are ambiguous with respect to if (and when) pregnant women should be included in clinical research and what renders their inclusion fair [30–34]. Many guidelines currently take the position that fairness implies the need to justify the exclusion of pregnant women from research unless there are compelling “scientific reasons” for their exclusion [33–35]. The default approach should be that researchers and RECs regard pregnancy as a case to avoid unfair exclusion [5, 34], particularly considering that real-life implications are that few drugs are approved for use during pregnancy, and a long period may elapses before teratogenic risk for prescription medications is evaluated [5, 34].

### The doctrine (principle) of double effect and permissibility of research on pregnant women

The doctrine (or principle) of double effect refers to the permissibility of an action that causes a serious harm, even to the extent of causing death, as an unintended consequence or side effect of actions that are intended to promote some good end [36, 37]. From the principle of double effect, it may sometimes be permissible to cause a harm as a side effect (or “double effect”) of bringing about a good result (or end) even when it would not be permissible to cause such harm as part of the intended means to bringing about the same good end [36, 37]. *Four* conditions should be satisfied for the application of the principle of double effect [37]: 1) *“The act itself must be morally good or at least indifferent”.* With regard to research in pregnancy, the research is needed to generate information in drugs to address illness in pregnant women, unborn children or newborns (making it a good end or result). Similarly, research is needed to evaluate the safety of many medications used in pregnancy, which are currently in use without adequate data on their effectiveness or safety profiles to the pregnant women or the unborn baby. The necessity to address this knowledge gap makes the research a moral action. 2) *“The agent may not positively will the bad effect but may permit it”.* If one could attain the good effect without the bad effect, one ought to do so, or ought to aim at doing so, such that the bad effect would often be indirectly voluntary [36, 37]. Therapeutic research in pregnancy may pose or even result in potential harms to the mother or unborn baby, even if this is not the intended outcome. Other non-therapeutic research may generate data on the state of pregnancy, which is beneficial to the mother or unborn baby. These include pharmacokinetic studies. 3) *“The good effect must flow from the action at least as immediately (in the order of causality, though not necessarily in the order of time) as the bad effect”.* Regarding research in pregnancy, the good effect (data that informs management of illness in pregnancy or newborns or data on prevention of illness in mothers and newborns) is produced directly by the action (by participation in the research), not by the bad effect (the potential risks and harms from research participation). The agent (the researchers) would not necessarily be using a bad means (research participation) to a good end (beneficial research outputs for the mother or baby). 4*) “The good effect must be sufficiently desirable to compensate for the allowing of the bad effect.”* The limited data on management of conditions in pregnancy or the fetus justifies the moral requirement for the research in pregnancy*.*

The principle of double effect is applicable to therapeutic research in pregnancy (especially some degree of) regarding “acceptable risk” to the fetus and mother. Research to address mothers or pregnant women’s or fetal illness which may be beneficial as the primary intended consequence may lead to untoward outcomes to the mother or unborn baby as an unintended consequence. The primary objective is not to induce this harm, yet this harm inevitably may occur. The challenge is that there is no clear definition of ‘acceptable risk’ to the woman or fetus and this uncertainty is perceived as a risk in itself, even when pregnant women may accept the uncertainty and risk in certain cases and may have no objection if invited to participate [5, 34]. The conditions provided for the principle of double effect include the explicit requirement that the bad effect is not the intended effect, even when it is foreseen to occur [36–39]. A person may licitly perform an action that one foresees will produce both a good effect and a bad effect provided that four conditions are verified [38]: *“that the action in itself from its very object be good or at least indifferent; that the good effect and not the evil effect be intended*; *that the good effect be not produced by means of the evil effect*; and that *that there be a proportionately grave reason for permitting the evil effect.”*

Worries about fetal safety often lead to clinicians or patients to avoid treatment or to undertreat some illnesses that continue or emerge during pregnancy. Yet failure to treat illness *can also* lead to significant harm to women and their fetuses, which harm may far outweigh any possible risks that might accompany use of medication during pregnancy [5, 34]. No greater than minimal risk is often considered a threshold for participation in research but there is uncertainty and practical challenges regarding how the concept should be applied in the context of research with pregnant women. This calls for development of a standard for no greater than minimal risk that should be applied when deciding on the inclusion/exclusion of pregnant women, that is, the potential risks that the pregnant woman in her particular situation would face, rather than referring to the general population of pregnant women as a whole [5, 34]. The justification adequate for causing a certain harm as a side effect might not be adequate for causing that harm as a means to the same good end under the same circumstances [37, 38]. This implies that not all research in pregnancy may be permissible, particularly non-therapeutic research. The researchers and research sponsors have the duty to consider not only to the potential risks of participation but also the potential risks of exclusion (that is the risks to the pregnant woman and fetus if she were not included in the research). Also, the research ethics committees (RECs) should consider both the potential risks and potential benefits of participation versus non-participation for not only the mother and fetus (that is to mothers with the problem under investigation), but also the benefit to society. RECs should require that researchers justify why pregnant women should be excluded from research if there is a possibility that the research can benefit the woman personally, the fetus, the newborn or pregnant women as a class of participants. Secondly, researchers should also justify why pregnant women should be included in case the study poses greater than minimal risk, even if the study benefits to mother and fetus are significant. In that case, the doctrine of double effect precondition of proportionality provides guidance that the benefits should not only be significant, but should outweigh the potential risks. Similarly, researchers should also justify the potential risks and harms that may result from research participation if the comparative potential benefits and potential risks for the fetus and pregnant woman differ.

### Implications of ethical considerations for research participation in pregnancy

In clinical research, there are several factors that lead to reluctance to include pregnant women in research. These include potential risk of harm to the pregnant women or her unborn baby, concerns about the physiologic complexity of women in general or pregnant women in particular, and higher potential for legal liability in case of adverse events from research related risks and herms [34]. Also, existing policies and regulations governing the inclusion of pregnant women in clinical research may be ambiguous, causing significant uncertainty and consequent barrier to research [34]. Besides, RECs may often exceed the regulatory requirements where the proposed research participants are pregnant women. From the Food and Drug Administration (FDA) guidance in the United States [40–42], research that involves pregnant women or fetuses should meet the following conditions: there should be adequate preclinical studies [43–45], including studies on pregnant animals, and clinical studies, including studies on nonpregnant women, to generate data for assessing potential risks to pregnant women and fetuses; the risk to the fetus should be caused solely by interventions or procedures that hold out the prospect of direct benefit for the woman or the fetus (or where there is no potential benefit, the risk to the fetus is not greater than minimal and the purpose of the research is to develop critical biomedical knowledge which cannot be obtained by any other means and the potential foreseeable risk is the least possible for achieving the objectives of the research. In addition, the research should have potential direct benefit to the pregnant woman or fetus. The conditions in the guidance satisfy the requirements for the doctrine of double effect.

In addition, there is additional guidance to reduce unnecessary potential risks and reduce conflict of interest [40–42]. The guidelines require that where research holds out the prospect of direct benefit solely to the fetus, the consent of the pregnant woman and the father is obtained in accord with the informed consent, and each individual who provided consent should be fully informed regarding the reasonably foreseeable impact of the research on health of the fetus or neonate. A further requirement is that individuals engaged in the research should have no part in decisions related to the timing, method, or procedures used to terminate a pregnancy or determining the viability of a neonate. No inducements should be offered to terminate a pregnancy. Thus from the above Food and Drug Administration guidance, research may be conducted where scientifically appropriate, preclinical and clinical studies on non-pregnant women provide an adequate basis for assessing potential risks to pregnant women and fetuses.

The ethical imperative for research has implications for ethical considerations for research participation, which include considerations of autonomy, beneficence and justice (or fair inclusion). Considering that cultural views on research participation in pregnancy can pose a significant barrier to the participation of pregnant women in research, RECs should lead strategies to engage communities as stakeholders to reconcile cultural norms and beliefs with the ethical and clinical rationale supporting the need and the justification for conducting research during pregnancy. To optimize potential benefits of research participation for pregnant women, there is also need to address the issue of communal consent for research. The autonomy of pregnant women may be compromised by cultural norms such as the need to seek permission from family members (spouse or in-laws) in the decision-making process. Where this is the cultural norm as happens in many settings, it is acceptable to integrate the consultation and engagement of other relevant family members in the consent and enrolment process for research participation. The final consent should, however, be given by the pregnant woman. Also, where possible, consent for research to be conducted later in pregnancy (or labor and puerperium) should be obtained as early as possible with the option to revisit it later during pregnancy. Strategies for beginning discussions about possible research participation earlier in pregnancy can mitigate some of the concerns about duress during later stages of pregnancy, labor or puerperium. Such information about possibilities and opportunities for research participation could be provided as part of health education talks during antenatal care.

### Implications for research on critical illness during pregnancy and newborns

Lastly, there is a challenge posed by participation in research during critical illness in pregnancy [5, 34]. Critically ill patients frequently undergo emergency treatment that affect their cognition (and capacity to comprehend disclosed information about clinical trial participation). Situations of uncertainty such critical illness and public health emergencies heighten the challenges associated with the inclusion of pregnant women in research. However, research in such situations ought to be permitted by RECs if the potential benefits justify the potential risks [46] and there is clinical equipoise [40]. The latter implies “a state of genuine uncertainty on the part of the clinical investigator regarding the comparative therapeutic merits of each arm in a trial.” Thus, from the principle of double effect, research should be deemed beneficial (potential to benefit the mother, fetus or both), there should be acceptable risk (there should be a favorable benefit-risk ratio), there is fair subject selection, the potential risks are minimized to the greatest extent that is feasible, and the option (decision-making) to participate is left to the pregnant women.

The doctrine (or principle) of double effect is usually invoked to explain the ethical permissibility of an action that causes a serious harm as a side effect of promoting some good end. The doctrine posits that if an action has two consequences (a double effect), the ethicality of the action depends on which of the consequences was intended, and which was a side effect of the intended action. This doctrine states that if doing something morally good has a morally bad side-effect, it may be ethically acceptable to do it providing the bad side-effect wasn’t the intended effect, and that this remains true even if one foresaw that the bad effect would probably happen, but would not be the main route through which the good outcome would result. Accordingly, sometimes it is ethically permissible to cause a harm as a side effect (or “double effect”) of bringing about a good result, if the harm is not intended to result into the good outcome, though it would not be permissible to cause such a harm as a means to bringing about the same good end. In the context of this article, this principle implies that a bad effect (research-related harms to the mother, unborn baby or newborn) may result as a side effect of research participation in clinical trials for pregnant women or newborns. The causation of such harms is not the direct intention of recruitment into research participation, even when it may be a foreseeable outcome of research on pregnancy and newborn complications.

Four conditions that must be fulfilled for the doctrine of double effect to be applicable. Firstly, *intrinsic permissibility*: act itself must be morally good or at least indifferent (neutral or independent of consequences). In the context of the article, research in pregnancy and newborn complications is necessary to generate information that will improve the management of these disorders by identifying or refining medications, procedures or practices used in pregnant women or newborns with complications. Secondly, *necessity:* the agent should not positively will or intend the bad effect, such that if the agent could achieve the good effect without the bad effect, the agent should do so. Thirdly, *intentionality:* the bad effect may be sometimes foreseeable but is neither primarily intended to occur nor is it intended to yield the good outcome or effect. The bad effect may be perceived as indirectly voluntary, that is, may be permitted, tolerated but is not intended. Also, the good effect must flow from the action at least as immediately (in the order of causality. Relating this precondition to clinical trials of pregnancy and newborn complications, the primary intention is to generate data that informs the management of the pregnancy or newborn complications. Considering that many of these may be critically-ill patients, some foreseeable risks and harms may occur, related to the illness or its management (that is, risks directly related to the illness or its management) or to research-related procedures (which may be a necessary component of the research process). The research-related harms may be foreseeable in research on pregnancy or newborn complications, as these are known to result from the complications themselves, the management used or the procedures involved. The bad effect may be perceived as indirectly voluntary, that is, may be permitted, tolerated but is neither intended to occur no to yield the good outcome or effect, may result as a side-effect of the actions that yield the good effect. Hence, the good effect must be produced directly by the action, not by the bad effect. Otherwise the agent would be using a bad means to a good end, which is not ethically permissible. The good effect should not arise out of the bad effect. Accordingly, the research-related harms may arise out of the intention to permit research participation for the mother’s or newborn’s benefit, but the primary intention is not to cause or induce the occurrence of these risks and harms. In any case, research = related procedures, including a rigorous selection criterion, may be employed to reduce such harms to the research participants. The good outcomes (research benefits) do not result from the bad outcomes (the research-related harms). If the good effect were the direct result of the bad effect, then the agent would intend the bad effect in pursuit of the good outcome.

Lastly, the condition of *proportionality* between the good and bad effect must be satisfied for the doctrine of double effect to be applicable to clinical trials of pregnancy and newborn complications. The good effect must be sufficiently desirable to compensate for the allowing of the bad effect, and the good effect must outweigh the bad effect. Thus, if the anticipated benefits from research participation far outweigh the potential risks, then the research on pregnancy or newborn complications may be ethically permissible. The ethicality of the action requires that the bad effect is permissible only if a proportionate reason compensates for permitting the foreseen bad effect to occur as a side effect of achieving the good effect. The social value of the research must outweigh any potential risks from research participation for the clinical research to be ethical [3]. Even then, the research procedures must ensure that the potential risks are reduced to the highest extent feasible or possible for enabling the research to continue to achieve a favorable benefits-risk ratio [3].

Research can generate information to develop, design, refine procedures, medications or practice that improve the management of pregnancy or newborn complications, thus there is intrinsic value in such research. Besides, research may generate data that contributes to reduction of the reticence of healthcare providers to provide pregnant women and newborns with access to potential benefits of research participation. Research involving pregnant women is necessary to provide women with effective treatment during pregnancy, to promote fetal safety (such as by avoiding the clinical use of drugs that may be harmful to the developing fetus), and to reduce avoidable harm from suboptimal care (such as from underdosing), hence the necessity for such research. Even when even when such research participation may involve potential foreseeable harms, this research participation is ethically permissible, because the primary intention is not to induce or cause such harms (hence they are not primarily intended to occur). The proportionality condition involves determination if the extent of the harm is adequately offset by the magnitude of the proposed or anticipated benefit. There is need to weigh the potential benefits and potential harms to the mother and fetus. The possibility of direct benefit (potential benefits) to the mother or fetus should be a prerequisite for participation, and particularly for studies that pose more than minimal risk to the participants, the concern should be on whether the potential benefits outweigh the potential harms.

To that end, potential risks to the pregnant women, the fetus or the newborn should be considered and weighed and where possible, mitigated against in order to make clinical trials of pregnancy and newborn complications ethically permissible. This is in line with the argument by Walzer [46] for an additional condition to the double doctrine effect - that agents minimize the foreseen harm even if this would involve accepting additional risk (to the mother, fetus or newborn) or foregoing some benefit (excluding potential participants from inclusion in research). The doctrine of double effect [38] distinguishes between *direct agency* and *harmful agency*. In direct agency, harm may come to some victims, at least in part, from the agent’s deliberate acts involving individuals in something in order to further one’s purpose precisely by way of their being so involved (agency where individuals are intentional objects). It also recognizes *harmful agency* in which either nothing is in that way intended for the victims or what is so intended does not contribute to their harm. The principle of double effect applies to outcomes of actions of well-intentioned agents who may cause a serious harm as a side effect of actions to bring about a good end of overriding moral importance (compared to the harm or bad effect) when it is impossible to bring about the good end without the risk of causing the potential harm [36–39]. This principle, I therefore argue, applies to therapeutic research in pregnancy and newborn complications. While harm may be cause d by research participation, the harm is not part of the investigators’ means to this good end (benefits of research participation to the individual or to generate scientific knowledge) and neither is it instrumentally intended to occur nor to bring about the good end.

My argument that it is ethically permissible to conduct clinical trials in participants with pregnancy or newborn complications is supported by the guidance from the Common Rule (45 CFR 46) [43]. For research involving pregnant women, fetuses, or neonates the institutional review board will approve the conduct of the research only if it finds that the research meets the regulatory criteria for approval addressed under the federal regulations at 45 CFR 46 Subpart B (45 CFR 46.204, “Research involving pregnant women or fetuses prior to delivery”; 45 CFR 46.205, “Research involving neonates”; 45 CFR 46.206, “Research involving, after delivery, the placenta, the dead fetus, or fetal material”. Where research fails to meet the criteria for approval addressed under 45 CFR 46.204, “Research involving pregnant women or fetuses prior to delivery”; 45 CFR 46.205, “Research involving fetuses after delivery”; or 45 CFR 46.206, “Research involving, after delivery, the placenta, the dead fetus, or fetal material,” the institutional review boards should find that that “the research presents an opportunity to understand, prevent, or alleviate a serious problem affecting the health or welfare of pregnant women or fetuses”; and" the research, if federally supported, will be submitted for review and approval by the Secretary, Department of Health and human services, in accordance with the provisions of 45 CFR 46.207. Research not otherwise approvable may even in itself present an opportunity to understand, prevent, or alleviate a serious problem affecting the health or welfare of pregnant women or fetuses, and in the context of this article, newborns with complications. Thus, such research may have intrinsic value and necessity. To assess the other preconditions of the double doctrine effect, (and in case the research study is not federally-funded), the institutional review boards have to co-opt obstetrician/ gynecology experts and an ethicist to recommend whether to approve the study as research that presents an opportunity to understand, prevent, or alleviate a serious problem affecting the health or welfare of pregnant women or fetuses (or in the case of the argument in this article, a serious problem of newborns). In that case, *intentionality* and *proportionality* are further assessed by the institutional review board.

Therefore, for the doctrine of double effect to be applicable to research on pregnancy and newborn complications, the following questions should be considered, which opponents may present as the counter-argument to the permissibility of clinical trials for pregnancy or newborn complications: Is the act in itself good or morally permissible? Is the bad effect unavoidable? Is the bad effect a means to achieve the good effect? Does the good effect outweigh the bad effect? Failure to appropriately answer the questions leads to criticism of the double effect doctrine, (and in this case, renders clinical trials of pregnancy or newborn complications ethically impermissible). In support of the counter-argument, individuals are responsible for all the anticipated consequences of their action. If an individual can foresee the two effects of the action, then they have to take the moral responsibility for both effects, rather than stating a claim that they did not have intentions for the bad effect. There is a view that intention is irrelevant, that it is sloppy morality to decide the rightness or wrongness of an act by looking at the intention of the person who carries it out. In support of such argument, some acts are objectively right or wrong, and that the intention of the person who does them is irrelevant. Therefore, it may be a misinterpretation to claim that the principle of double effect shows that agents may permissibly bring about harmful effects provided that they are merely foreseen side effects of promoting a good end. A second misinterpretation concerns the permissibility of causing a harm as a merely foreseen side effect of pursuing a good end and impermissibility of causing the same kind of harm as one’s end. Some philosophers argue that if an agent recognizes that a certain consequence will inevitably follow from a contemplated action, then in performing the action the agent must be intending the consequence. Others argue that the doctrine fails to delineate a practicable criterion for marking off the intended from the merely foreseen. It is widely accepted that it is wrong to aim to produce harm to someone as an end. The principle presupposes that agents do not aim to cause morally grave harms as an end, and guides decisions about causing harm in pursuing a morally good end.

A third misinterpretation of double effect is the false assurance that agents perform their acts provided that their ultimate aim is a good one that is ordinarily worth pursuing (has high value), as long as the proportionality condition is satisfied and the harm is minimized. This may not be sufficient. For instance, it is also true that causing the harm should not be implicated as part of the agents’ means to this good end, that it should not be something or act that is instrumentally intended to bring about the good end. Therefore, it would be ethically impermissible to permits acts that cause certain kinds of harm (just) because those harms were not the agent’s ultimate aim or were regretted rather than welcomed. In the context of my argument, potential research-related harms produced regretfully and only for the sake of producing a good end (gaining individual or societal benefits from research participation) are prohibited by double effect doctrine. This is because such research-related harms would be caused as part of the agent’s means to realizing the good end (the benefits from pregnant women or newborns’ research participation in clinical trials). Even then, some argue, the distinction between intended and merely foreseen consequences may not have moral significance.

As counter claim to the above criticism of the application of the doctrine of double effect, the four preconditions have to be met if the action in question is to be morally permissible. First, that the action contemplated be in itself either morally good or morally indifferent; second, that the bad result not be directly intended; third, that the good result not be a direct causal result of the bad result; and fourth, that the good result be “proportionate to” the bad result. Where these conditions are met, the action under consideration is morally permissible (in this case permitting clinical trials of pregnancy or newborn complications) despite the bad result (potential harms to the research participants or the unborn baby). Besides, the doctrine is directed at well-intentioned agents, who in seeking to pursue a morally right act, question whether they may cause a serious harm in pursuit of their efforts to bring about a good end, especially a good end of overriding moral importance, when it is impossible to bring about the good end without the harm as a side effect. Also, applications of double effect always presuppose that some kind of proportionality condition has been satisfied. Similarly, formulations of double effect should require that the value of promoting the good end outweigh the disvalue of the harmful side effect. Even then, the double effect doctrine is silent about situations or contexts where a small harm might permissibly be brought about as a means to a good end.

In addition, most acts have good and bad outcomes. The bad outcome may be foreseen but not intended. The act itself that yields bad outcomes may not be intrinsically wrong. The good outcome may be produced directly by the act and not by the bad effect. The good outcome may be sufficiently desirable to compensate for the bad outcome. The double effect doctrine therefore shows that an act with a negative outcome may be acceptable as long the intention was not to cause the negative outcome. Besides, absolutist prohibitions are about deliberate acts. The doctrine is about what one does not what one allows, and acknowledges that actions can have multiple outcomes or effects, some intended, other unintended. This argument is supported by observations that there are always times when we have moral dilemma where cannot do good without bad or harmful consequences. It is always wrong to do a bad act intentionally even if the intention is to bring about something good. However, it is sometimes right to do something good even if we know that something bad may result as unintended consequences. Thus, an action which in itself is good, having two effects, an intended (even if otherwise not easily attainable) good effect and a foreseen bad effect is licit, provided there is proportion between the intended good and permitted bad outcome. When there is a clash between the universal norms of “do good” and “do no evil,” the question arises as to whether the obligation to avoid evil necessitates one to abstain from doing the good thing so as to prevent or avoid a merely permitted though foreseen concomitant evil. One need not always need to abstain from the good action that has foreseeable bad effects, depending on other certain moral criteria explained by the principle of double effect. Thus, right-intention and proportionality-intention matter if the doctrine of double effect is to be licitly applied. Thus when an act intended to have good effects can only achieve this at the risk of causing a bad or harmful effect, this ethically may be permissible, if the action itself is good, the intention is solely to produce the good effect, the good effect is not achieved through the bad effect, and there is sufficient reason to permit the bad effect.

## Conclusion

The double effect doctrine may be invoked to argue for the ethical permissibility of conducting clinical trials for pregnancy or newborn complications. Invoking the double effect doctrine involves making a comparative judgment, that is, asserting a claim that a harm that might permissibly be brought about as a side effect in promoting a good end could not permissibly be brought about as a means to the same good end, and the good end cannot be realized without some unintended harmful side effect, but the harm should be much less in proportion to the anticipated gains of the action. Hence, an investigator’s intentions, motives, and attitudes are important considerations in determining the ethical permissibility of a course of action (in this case, permitting the involvement of pregnant women or newborns with complications in research), and not merely the consequences of the action (the foreseen societal, scientific or participant gains from research participation and the foreseen or foreseeable potential research-related harms). In the context of this article, the prospective investigators’ motives and intentions, should justifiably indicate that they have considered, and have contingency plans in place, to reduce potential harms that may result from research participation in clinical trials of pregnancy or newborn complications. Both the nature-of-act and means-to-end act matter. A harmful effect of research participation is morally permissible provided it was not intended, arises merely as a side effect of a beneficial action, the harmful effect was not the means of achieving the beneficial action, and the bad or harmful effect is not out of proportion to the good effect. Thus, one should not will to act with evil intentions. One ought to intend the good effect, but should (be ready to) tolerate the bad or harmful effect. The good effect should not arise a result of the bad effect and there should be a proportionate reason for that harmful or bad effect to have occurred. Therefore, the bad intent should not be the means to a good end. A good effect that is intended may come along with it a bad effect, which may be reasonably foreseen, yet this does not render the act impermissible if the bad effect is not intended, does not arise from the bad effect, or is not out of proportion to the good effect.

## Data Availability

Not applicable.

## Abbreviations

FDA: Food and Drug Administration
IRB: Institutional review boards
PK: Pharmacokinetics
RCTs: Randomized clinical trials
REC: Research Ethics Committees
